# A CRISPR-Cas9 screen shows the combination efficacy of lenvatinib plus epidermal growth factor receptor inhibitors for treatment of liver cancer

**DOI:** 10.20892/j.issn.2095-3941.2021.0520

**Published:** 2021-10-28

**Authors:** Yuchen Guo, Yangyang Zhou, Wenxin Qin

**Affiliations:** 1 State Key Laboratory of Oncogenes and Related Genes, Shanghai Cancer Institute, Renji Hospital, Shanghai Jiao Tong University School of Medicine, Shanghai 200032, China

Recently, in cooperation with the Netherlands Cancer Institute, we demonstrated that epidermal growth factor receptor (EGFR) activation limited the response of liver cancer to lenvatinib. The original article was published in *Nature* as a cover story, which resulted in considerable attention from major scientific journals. Here, we were invited by *Cancer Biology & Medicine* to provide comments on preclinical and clinical findings about the combination of lenvatinib plus EGFR inhibitors as a promising strategy for hepatocellular carcinoma (HCC) treatment.

Primary liver cancer represents the sixth most common malignancy and the third leading cause of cancer-related mortality worldwide, with an estimated 906,000 new cases and 830,000 deaths in 2020^1^. According to the latest GLOBOCAN 2020 statistics on cancer incidence and mortality from the International Agency for Research on Cancer, 45.3% of new liver cancer cases and 47.1% of liver cancer deaths are estimated to occur in China. The occurrence of liver cancer in Europe and the United States mainly results from an increase in nonalcoholic-associated steatosis and obesity^2,3^. HCC accounts for 85%−90% of all cases of primary liver cancer. However, only 20% of HCC patients are diagnosed during their early stages. The majority of HCC patients are usually diagnosed during advanced stages, resulting in few treatment options. The 5-year survival of HCC patients is less than 18%^4^. Therefore, identifying effective therapeutic strategies for patients with advanced HCC is urgent. Unfortunately, most prevalent gene mutations in HCC patients, such as *TP53*, *CTNNB1*, and the *TERT* promoter cannot be treated with drugs^5^. The current clinical standard for advanced HCC involves multi-kinase inhibitors, such as sorafenib, lenvatinib, and regorafenib, and they provide only modest survival benefits to patients.

Lenvatinib is an oral multi-kinase inhibitor that targets vascular endothelial growth factor (VEGF) receptors 1−3, fibroblast growth factor (FGF) receptors 1−4, platelet-derived growth factor (PDGF) receptor α, “rearranged during transfection” (RET), and receptor tyrosine kinase (KIT). The approval of lenvatinib as the first-line standard therapy for advanced HCC is based on the results of the randomized, open-label, multinational, non-inferiority phase III REFLECT trial in patients with unresectable HCC^6^. In the REFLECT trial, lenvatinib has been shown to be similar to sorafenib for overall survival (OS). Because sorafenib has been approved during the last decade, in November 2018, with its launch in China, lenvatinib has become another new drug for the standard therapy of advanced HCC. Although lenvatinib has been associated with significant improvements in the objective response rate (ORR), when compared with sorafenib (24.1% *vs*. 9.2%; *P* < 0.0001), the majority of HCC patients received limited benefits from lenvatinib. The lack of response can be driven by either intrinsic resistance or rapidly acquired resistance to this drug. There is therefore an urgent need to identify the mechanism of drug resistance as well as identify combination strategies to improve the clinical benefits of lenvatinib for HCC patients (**Figure 1**).

**Figure 1:**
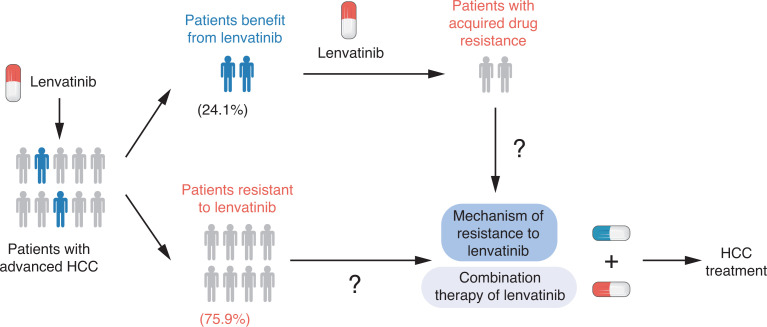
Lenvatinib in the treatment of hepatocellular carcinoma patients.

## Synergistic effects of lenvatinib and EGFR inhibition on HCC cells

**Figure 2:**
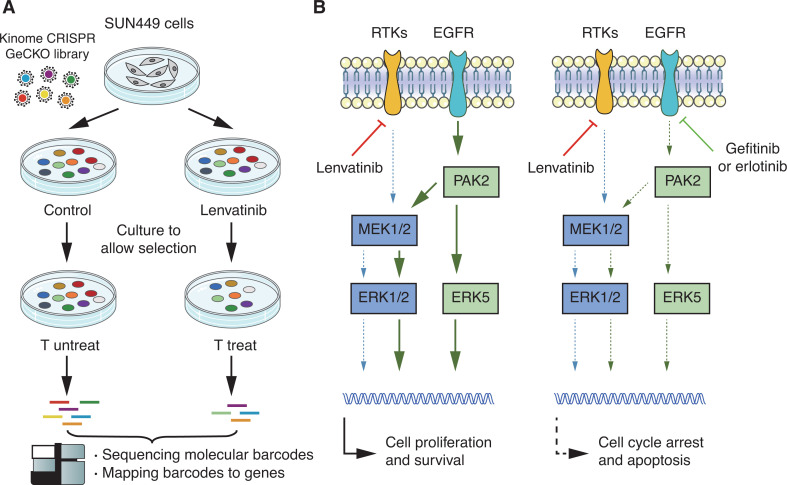
Epidermal growth factor receptor (EGFR) identified as a synthetic lethal target of lenvatinib in hepatocellular carcinoma cells. (A) Schematic outline of the synthetic lethal screen using lenvatinib. (B) Activation of the EGFR-PAK2-ERK5 axis by lenvatinib is blocked by EGFR inhibitors, leading to synergy.

By using synthetic lethality screening that targets the human kinome using CRISPR/Cas9 technology, Jin et al.^7^ reported that depletion of EGFR significantly increased the sensitivity of HCC cells to lenvatinib (**Figure 2A**). The majority of HCC cell lines were resistant to lenvatinib; however, HCC cells expressing high levels of EGFR showed strong synergistic effects of lenvatinib plus EGFR inhibitors on cell growth. Mechanistically, inhibition of key receptor tyrosine kinases such as FGFR by lenvatinib leads to feedback activation of EGFR and downstream cascades of the PAK2-ERK5 and MEK1/2-ERK1/2 signaling pathways, thus maintaining survival and proliferation of HCC cells in the presence of lenvatinib. A combination of lenvatinib and EGFR inhibitors (gefitinib or erlotinib) synergistically blocks both the FGFR-mediated and EGFR-mediated ERK/MAPK signaling pathways, leading to cell cycle arrest or apoptosis of HCC cells^7^ (**Figure 2B**).

## Lenvatinib synergizes with EGFR inhibitors by abrogating FGFR signaling

Currently, toxicity and the pharmacokinetics of EGFR inhibitors have been known for their wide clinical use in cancer patients. In previous clinical trials, erlotinib as an EGFR inhibitor showed good tolerance but extremely limited treatment efficiency in HCC patients^8,9^. Additionally, the results of a large-scale phase III randomized double-blind placebo-controlled trial showed that a combination of sorafenib plus erlotinib did not result in significant clinical benefits to HCC patients^10^. Consistent with this previous report, Jin et al.^7^ also found that EGFR inhibitor minimally sensitized HCC cells to sorafenib. Similar to lenvatinib, sorafenib is a multi-kinase inhibitor that targets VEGFR1-VEGFR3, PDGFRα, KIT, and RET, but does not inhibit FGFR family members. Jin et al.^7^ therefore speculated that lenvatinib potentially synergized with EGFR inhibitors by abrogating the FGFR signaling pathway. This hypothesis has been confirmed by the synergistic effects of FGFR-specific inhibitors (BGJ398 and AZD4547) plus an EGFR inhibitor^7^.

## Combination therapy of lenvatinib plus EGFR inhibitors in animal models with HCC

The combination therapy of lenvatinib plus EGFR inhibitors has been investigated *in vivo* in both HCC xenografts and genetically engineered mouse models with orthotopic liver cancer. The results showed that a combination of lenvatinib plus EGFR inhibitors was very beneficial for the treatment of HCC patients with high protein levels of EGFR^7^. Immunotherapy has recently shown great potential in the treatment of liver cancer. Immune checkpoint blockade (ICB) mono-therapy has been used as a second-line treatment of advanced HCC since 2017, and clinical use of ICB combined with anti-angiogenesis drugs was used for the first-line treatment of advanced HCC since 2020^11^. Since these findings, the use of immunotherapy combined with targeted therapies as a treatment for HCC patients has generated intense interest. Jin et al.^7^ reported that compared with lenvatinib mono-therapy, lenvatinib plus gefitinib increased the infiltration of NK and CD8^+^ T cells, while it decreased the number of tumor-associated macrophages *in vivo*. These results indicated that combination therapy using EGFR inhibitors plus lenvatinib remodeled the immune microenvironment of HCC, which provided a novel clue for future immunotherapy combinations^7^.

## Perspectives in combination therapy for HCC

**Figure 3:**
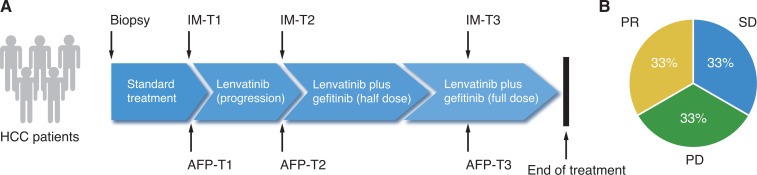
Responses of hepatocellular carcinoma (HCC) patients to combination therapy. (A) The study design of the clinical trial of HCC patients resistant to lenvatinib. (B) Clinical responses of 12 HCC patients after combination therapy of lenvatinib plus gefitinib. PR, partial response; SD, stable disease; PD, progressive disease.

Based on preclinical findings, Jin et al.^7^ registered a phase I clinical trial (trial identifier: NCT04642547) to investigate the safety and therapeutic efficiency of lenvatinib combined with gefitinib in lenvatinib-resistant HCC patients. Only HCC patients with high EGFR expression levels were recruited in this study. Published data from 12 advanced HCC patients who were unresponsive to lenvatinib mono-therapy showed that 4 out of 12 patients had a confirmed partial response (PR) after combination therapy of lenvatinib plus gefiitnib, indicating a ORR of 33% (**Figure 3**). It is noteworthy that another phase I clinical trial has been initiated at the Memorial Sloan Kettering Cancer Center to evaluate the clinical efficacy of a combination of the EGFR monoclonal antibody, cetuximab, plus lenvatinib for the treatment of head and neck squamous cell carcinoma (HNSCC) patients. As reported at the American Society of Clinical Oncology (ASCO) conference in 2020, of the 9 HNSCC patients treated with combination therapy of cetuximab plus lenvatinib, 6 patients had a PR with 67% of ORR^12^. These results strongly suggested that a combination of lenvatinib plus EGFR inhibitors may have clinical benefits for HCC, as well as for other types of human cancers. Therefore, it is important in future studies to evaluate the efficacies of combination treatment strategies of lenvatinib plus EGFR inhibitors in other tumor types.
